# The Potential Role of Cytokines and Growth Factors in the Pathogenesis of Alzheimer’s Disease

**DOI:** 10.3390/cells10102790

**Published:** 2021-10-18

**Authors:** Gilbert Ogunmokun, Saikat Dewanjee, Pratik Chakraborty, Chandrasekhar Valupadas, Anupama Chaudhary, Viswakalyan Kolli, Uttpal Anand, Jayalakshmi Vallamkondu, Parul Goel, Hari Prasad Reddy Paluru, Kiran Dip Gill, P. Hemachandra Reddy, Vincenzo De Feo, Ramesh Kandimalla

**Affiliations:** 1 School of Public Health, The University of Texas Health Science Center (UTHealth), Houston, TX 77030, USA; gbrain17@gmail.com; 2 Advanced Pharmacognosy Research Laboratory, Department of Pharmaceutical Technology, Jadavpur University, Kolkata 700032, India; saikat.dewanjee@jadavpuruniversity.in (S.D.); pratik.chakraborty88@yahoo.com (P.C.); 3 Department of Medicine, Mahatma Gandhi Memorial Hospital, Warangal 506007, India; cvalupadas@gmail.com; 4 Department of Medicine, Kakatiya Medical College Superspeciality Hospital, Warangal 506007, India; 5 Orinin-BioSystems, LE-52, Lotus Road 4, CHD City, Karnal 132001, India; a.chaudhary-lifescience@outlook.com; 6 Department of Biochemistry, GITAM Institute of Medical Sciences and Research, Visakhapatnam 530045, India; kolli.kalyan@gmail.com; 7 Department of Life Sciences, Ben-Gurion University of the Negev, Beer-Sheva 84105, Israel; ushuats@gmail.com; 8 Department of Physics, National Institute of Technology, Warangal 506004, India; vlakshmij@gmail.com; 9 Department of Biochemistry, Maharishi Markandeshwar Institute of Medical Sciences & Research, Mullana, Ambala 133207, India; parul006@gmail.com; 10 Department of Biotechnology, Sri Krsihnadevaraya University, Anantapur 515003, India; hareereddy@gmail.com; 11 Department of Biochemistry, Research Block-A, Posgraduate Institute of Medical Education & Research (PGIMER), Chandigarh 160012, India; kdgill2002@yahoo.co.in; 12 Department of Internal Medicine, Texas Tech University Health Sciences Center, Lubbock, TX 79430, USA; hemachandra.reddy@ttuhsc.edu; 13 Department of Neuroscience and Pharmacology, Texas Tech University Health Sciences Center, Lubbock, TX 79430, USA; 14 Departments of Neurology, School of Medicine, Texas Tech University Health Sciences Center, Lubbock, TX 79430, USA; 15 Public Health Department of Graduate School of Biomedical Sciences, Texas Tech University Health Sciences Center, Lubbock, TX 79430, USA; 16 Department of Speech, Language and Hearing Sciences, School Health Professions, Texas Tech University Health Sciences Center, Lubbock, TX 79430, USA; 17 Department of Pharmacy, University of Salerno, 84084 Fisciano, Italy; 18 Applied Biology, CSIR-Indian Institute of Technology, Uppal Road, Tarnaka, Hyderabad 500007, India; 19 Department of Biochemistry, Kakatiya Medical College, Warangal 506007, India

**Keywords:** Alzheimer’s disease, cytokines, chemokines, neuroinflammation, neurotrophic factors, pathophysiology, blood brain barrier, mild cognitive impairment, brain health, therapeutics

## Abstract

Alzheimer’s disease (AD) is one of the most prominent neurodegenerative diseases, which impairs cognitive function in afflicted individuals. AD results in gradual decay of neuronal function as a consequence of diverse degenerating events. Several neuroimmune players (such as cytokines and growth factors that are key players in maintaining CNS homeostasis) turn aberrant during crosstalk between the innate and adaptive immunities. This aberrance underlies neuroinflammation and drives neuronal cells toward apoptotic decline. Neuroinflammation involves microglial activation and has been shown to exacerbate AD. This review attempted to elucidate the role of cytokines, growth factors, and associated mechanisms implicated in the course of AD, especially with neuroinflammation. We also evaluated the propensities and specific mechanism(s) of cytokines and growth factors impacting neuron upon apoptotic decline and further shed light on the availability and accessibility of cytokines across the blood-brain barrier and choroid plexus in AD pathophysiology. The pathogenic and the protective roles of macrophage migration and inhibitory factors, neurotrophic factors, hematopoietic-related growth factors, TAU phosphorylation, advanced glycation end products, complement system, and glial cells in AD and neuropsychiatric pathology were also discussed. Taken together, the emerging roles of these factors in AD pathology emphasize the importance of building novel strategies for an effective therapeutic/neuropsychiatric management of AD in clinics.

## 1. Introduction

Neurodegeneration has been a puzzle gradually elucidated by the progress of ample research and the investigation of dementia and progressive cognitive decline. Dementia which is marked by the affliction of Alzheimer’s disease (AD), is understood as the decline in memory and other fundamental cognitive functions. AD is the most commonly occurring neurodegenerative disease in the world. AD has been extensively characterized by the gradual decline of neuronal health. Neurotoxins, TAU protein neurofibrillary tangles, amyloid-beta (Aβ) plaque accumulation in mature neuron phenotypes [1,2,3,4,5], mitochondria dysfunction (fusion-fission imbalance) [6,7], and neuroinflammation collectively involves in neurodegeneration in AD [8,9,10,11]. Mitochondrial dysfunction results in the accumulation of harmful reactive oxygen species (ROS), which subsequently trigger CNS apoptotic decline [7]. Neuroinflammation is mainly governed by the actions of cytokines, chemokines, and growth factors, which play key roles in neurodegeneration [8,9,10]. These aberrancies have been widely reported as fundamental hallmarks of AD and its pathological quantification [12,13].

Cytokines are non-structural proteins within the molecular weight range of 8000–40,000 Da. They can be described as inflammatory peptides aiding the immune defense response. The majority of nucleated cells are capable of synthesizing cytokines but they are predominantly produced by macrophages/microglia and lymphocytes [14]. These cells can in turn also respond to and interact with cytokines. Cytokines can be grouped into certain classes based on their biological activities which could be pro-inflammatory or anti-inflammatory. The biological activities of cytokines are vast and range from cell proliferation to apoptosis and from cell differentiation to inflammatory responses. Cytokines are also termed lymphokines since they are primarily involved in the differentiation of different types of T lymphocytes *viz.* T helper cells, and T regulatory cells from undifferentiated cells [15]. Many of these proteins, for example, interleukins (ILs), interferons (INFs), tumor necrosis factors (TNFs), and certain growth factors are produced by neurons and glial cells of the brain in the event of neuroinflammation. Levels of IL-1α, IL-1β, IL-6, TNF-α, IFN-α, macrophage colony-stimulating factors (MCSFs), IFN-α and IL-8 receptor type B are enhanced in blood and cerebrospinal fluid (CSF) in AD patients. Nerve growth factors (NGF), growth-promoting properties of APP, vascular endothelial growth factor (VEGF) also play vital roles in the pathophysiology of AD. Growth factors are proteins by nature and support the survival of cells within the nervous system. Moreover, they are vital players for the proper development of the brain. In the CNS and PNS, they stimulate axonal growth and regulate the growth of different kinds of cells.

AD is named after German psychiatrist and neurologist Alois Alzheimer [16]. In 1906, the doctor noted some peculiar findings in the brain of a patient who passed away after suffering from memory loss, disorientation, paranoia, and unpredictable behaviors. AD causes a gradual decline in cognitive processes starting with mild cognitive impairment (MCI) reaching a stage of severe irreversible loss of cognition and functionality (Table 1). AD, by nature, is an insidious, progressive, and degenerative disorder. Given the fact that the improvements in medical science considerably improve the quality of life and increase life expectancy in afflicted individuals, a longitudinal study that began with a cohort of normal subjects revealed a higher incidence of AD in women compared to men with the largest incidence in age group ≥ 85 (95% CI 5.01 to 8.38) [17], and epidemiological studies of the prevalence of AD show a positive correlation with increasing age [18]. AD invariably starts from the hippocampus (responsible for new memory generation) making anterograde amnesia a primary symptom of the disease. As neurofibrillary tangles start to spread outward towards the frontal lobe, dementia is followed by progressive speech problems, mood imbalance, and inability in decisions making [19]. Several genes including the senilins, SORL1, APP, and ApoE4 were found to play crucial roles in the onset and progression of AD [19]. Early AD onset is generally familial, while late AD onset is largely related to SORL1. From the viewpoint of pathophysiology, AD is characterized by intracellular neurofibrillary tangles and extracellular senile plaques. Assessment of Instrumental activities of daily living in a geropsychiatry clinic revealed that impairment and memory loss was higher in patients with mild cognitive impairment (MCI) (*n* = 66) compared to control subjects (*n* = 61) [20]. During the course of AD progression, individuals begin to experience cognitive decline prior to clinically diagnosed MCI. In a longitudinal study by Cloutier et al., compared to healthy controls who did not progress to an MCI diagnosis, individuals who were previously healthy and later expressed cognitive impairment showed different patterns of impairment years prior to an MCI diagnosis and escalating severity of decline was observed over time [21]. The incidence of neuropsychological decline constituting memory loss, episodic cognitive decline, and executive function decline 12 years before MCI diagnosis indicate that neuroinflammation is present in neurodegeneration that leads to AD prior to diagnosable MCI [22]. Brain hypometabolism map PET scan analysis corroborated that the activation of microglial regional clusters in the brains of individuals is predominantly involved in the transition from healthy status to dementia [23], which divulges the involvement of inflammation in neurodegeneration leading to AD.

Identification and elucidation of the roles of cytokines and their co-associating factors, such as growth factors, in the immune system and in response to the pathogenesis of AD, is a key step to explore their potentials for therapeutic interventions. This review aims to analyze research data, prior AD-related research, and affiliations between connected fates of inflammatory and immune responses of AD, to help identify the role of cytokines and key growth factors implicated in AD.

## 2. Immune Response in AD: Role of Cytokines

Cytokines mediate cell functioning, cell signaling behaviors, and neuro-immune activity and are classified by the actions that they solicit. During AD immune response, such cytokines include pro-inflammatory cytokines, anti-inflammatory cytokines, and cytokines that are known to inhibit virus replication. These cytokines can activate macrophages, B-cells, T-cells, and mast-cells and constitute a cytokine network in the brain. In AD, certain cytokines are involved in the immune responses that precede and stimulate the actions of other cytokines in the innate neuroimmune inflammatory reactions. It was observed in AD consequent of aberrant pathologies in the brain and concomitant to CNS insults that include neurotoxicity, accumulation of Aβ senile plaque, and TAU pathologies (Table 2). IL-1α containing plasmids were analyzed in IL-1 cDNA clones by the hybrid selection of biologically active mRNA that resulted in abundant IL-1 expression in LPS-stimulated macrophages [24].

Of the classes of cytokines that are implicated in AD, specialized groups of cytokines are differentiated by the availability of their receptors expressed on the cell surface of implicated cell types and the condition of the genes that regulate these receptors. Cytokines play a major role in routine neurological activities of the CNS in the transfer and reception of chemical cues that confer instructions on cell actions and reactions. Chemotactic cytokines that function as chemoattractant cytokines, such as IL-8 and IP-10/CXCL10 may experience N-terminal proteolytic alteration after being secreted.

### 2.1. Immune System in AD and Cytokines

At the beginning of neurodegeneration, the immune reactions trigger macrophage activation (predominantly M2 and sometimes M1) [25]. These macrophages secrete chemical messengers in interneuronal communications and develop autoimmune neurotoxicity including those reactions that lead to neuroinflammation and escalation of AD. The immune system employs cytokines, which play a major role in immune responses following the activation of microglia in the pathology of AD. Cytokines determine the mechanisms and reactions that take place in the immune system in response to abnormal changes in the neurons. These trigger the recruitment of other defensive cells including neutrophils and macrophage progenitor cells.

**Figure 1:**
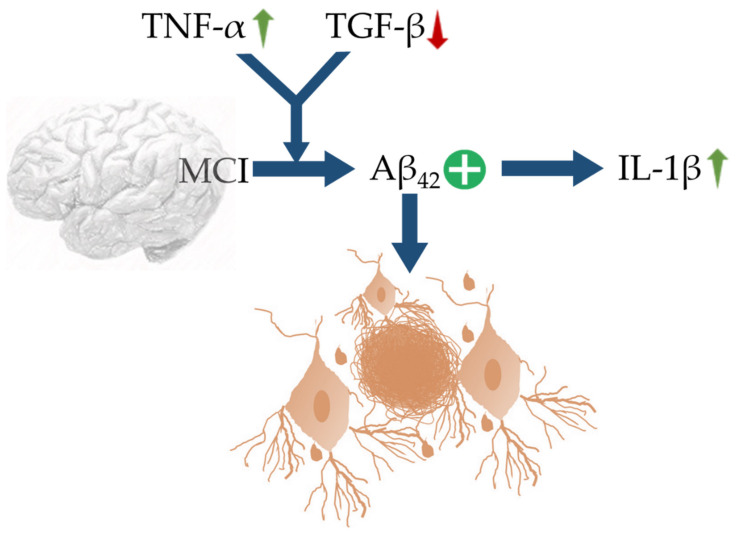
Schematic representation of MCI, linked with up-regulation of TNF-α and decrease in TGF-β characterized by upregulation of IL-1β and Aβ42 expressions. The blue arrows (**↑**) indicate downstream cellular events, upward green arrows (**↑**) indicate upregulation, downward red arrow (↓) indicates down-regulation, and plus sign (**+**) indicates enhanced activity.

In the case of AD, Aβ originating from APP trigger the rest of the pathologies. Aβ outside the neurons and neurofibrillary tangles inside the neurons make up for the development of AD [77,78]. Aβ further produces immune response activating complement systems. In CNS, the immune system is programmed to functionally respond to pathological changes such as those presented by the progression of AD [25]. The immune system activation observed in AD is labelled as neuroinflammation [79]. Herein, misfolded and aggregated proteins i.e., Aβ act through danger-associated molecular pathways (DAMP) to bind pathogen recognition receptors such as CD14, CD36, α6β1, integrin, and toll-like receptors (TLRs) [80]. These, in turn, control functions of ROS, NO, IL-1β and TNF-α. It has been experimentally shown that, contrary to antiquated conclusions about neuroinflammation, observed in MCI, early, and late AD onset are initiating events predominantly driven by the CNS resident immune cells, such as microglia and perivascular myeloid cells [79]. An up-regulation of TNF-α with concomitant suppression in TGF-β synergize Aβ42 deposition in MCI, which further trigger neuroinflammation via recruiting IL-1β (Figure 1). Genetic variants and transcription factors also determine the expression of activated microglia in the pathological environment. Damaging or degenerating neurons give off signals acting as a form of microglial control switch that stimulates microglia which could become cytotoxic from the reactive intermediates solicited such as pro-inflammatory cytokines [81]. In response to a change in homeostasis, microglia must first be activated, changing it from a static to a primed state. Changes in infiltrating monocytes that support CNS immune response in the parenchyma and neuronal progenitor granule crossing the BBB might be a hallmark for early detection of AD and propensity of inflammatory response and neurodegeneration [82]. Asymmetrical changes in serum and plasma levels of cytokines may indicate changes in early cytokine levels widely reported in macrophage precursor cells that may confer a greater risk of developing neurodegeneration and abnormal macrophage morphology.

### 2.2. Roles of Cytokines in Autophagy

Aβ burden has been revealed to be positively correlated with age [51] and exacerbated by oxidative stress, such as GAPs that promote the generation of ROS [54] that perturb brain health [83,84,85]. Glycation end products that confer oxidative stress in AD, which was found to be heavily associated with ApoE in its dimeric form greater than its monomeric form at Aβ accumulation site [55]. An increase of ApoE can lower the Aβ_40–42_ turnover rate on greater cognitive decline in AD [57]. The same has also been found to negatively influence or disturb autophagy by disrupting autophagosome formation [59]. This, in turn, leads to greater deterioration of neuronal health in AD pathology. Autophagy is critical for Aβ clearance and important in the maintenance of homeostasis in the CNS. In concert with dysfunction of autophagy, mitophagy was observed to express excessive fragmentation, decline in synaptic integrity [60], and an imbalance of mitochondrial dynamics [61,62]. Dysfunction of autophagy/mitophagy indicates a notable neuroinflammatory pathology and involvement of cytokines. IL-1β and IFN-γ (which are known to be expressed in AD pathogenesis) exposure to primary rat β-islet cells hindered autophagy resulting in cell apoptosis [64] and additionally, IL-1β was reported to modulate microglia autophagy in LPS cultures in the presence and absence of Aβ42 [67,86]. This evidence suggests that IL-1β and IFN-γ maintain control of inflammation in AD via lysosomal pathway and initiation of phagophore assembly.

### 2.3. Cytokines and BBB

There exists a definite correlation between brain cytokine levels and neuropsychiatric disorders. Right at this point, selectivity, and integrity of BBB to cytokines become important. Cytokines are pleiotropic, hence their release, unlike hormones has more complicated effects on the regulation of neurotransmission. Cytokines can cross BBB, activate free calcium, and by disrupting the compartmental model of brain calcium homeostasis, compromise the integrity of BBB [87]. Many cytokines can pass through BBB directly [88]. Interestingly, glial cell-derived neurotrophic factors bypass the BBB by simple diffusion through circumventricular organs. Whereas passage of IL-1α, IL-6, and TNF-α involves saturable influx transport through retrograde axonal transport system [87,89]. TNF-α, a downstream cytokine of chemokine IP10, decreases tight junction proteins leading to the destruction of endothelial tight junctions of BBB to affect its permeability [90]. On the other side, inhibition of mTOR hyperactivity has been reported to protect the integrity of BBB in AD [91]. Therefore, BBB dysfunction brings about early aging in the brain paving the way for AD and other neurodegenerative disorders.

## 3. Role of Cytokines and Chemokines in Neuropsychiatry

**Figure 2:**
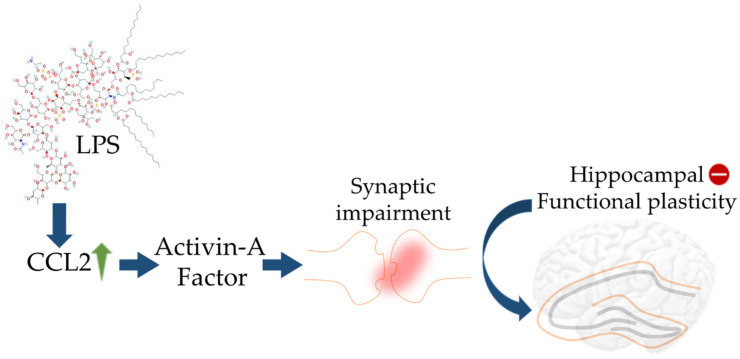
Schematic diagram showing impact of LPS on elicited CCL2 activity in turn leading to aberrant hippocampal plasticity. The blue arrows (**↑**) indicate downstream cellular events, upward green arrow (**↑**) indicates upregulation, and minus sign (**−**) indicates decreased activity.

The study of cytokines to understand the pathophysiology of neuropsychiatric disorders such as dementia, anxiety, and delirium has been pioneered by Dr. M. Maes who first linked vegetative symptoms with enhanced presence of IL-1, IL-6, and haptoglobin [87,92]. Chemokines regulate the migration of microglia and the recruitment of astrocytes to the sites of inflammation. Cytokines may act in an autocrine, paracrine, or endocrine fashion and generally are upregulated at sites of Aβ plaques. Aβ peptides mediate cell mediators, such as monocytes are also responsible for the generation of IL-8, monocyte chemoattractant protein 1 (MCP1), MIP1α, and MIP1β. LPS stimulates astrocytes to secrete cytokines including IL-6 and TNF-α, activates astrocytoma cells to secrete IL-6 and IL-8 and monocytes to secrete IL-8 under the influence of Aβ peptides [93]. Synergistic activity of cytokines has also been reported along with Aβ peptides e.g., TNF-γ synergizes with Aβ to enhance secretion of TNF-α and reactive nitrogen species [39]. IL-1β displays pro-inflammatory actions via MEK 1/2, JNK-activated α-secretase cleavage and upregulated a disintegrin and metalloprotease (ADAM)-17/TNF-α converting enzyme (TACE) pathway to increase sAPPα secretion [94]. On the contrary, IL-1β can also serve as an anti-amyloidogenic factor by decreasing sAPPβ and amyloidogenic Aβ fragment levels by reducing β-secretase cleavage [95]. It was also suggested that increased Aβ clearance by microglia in models of sustained IL-1β neuroinflammation could involve Th2 cytokines, such as IL-4 [30]. Moreover, a feedback signalling loop between Aβ and IL-1β was also proposed in which Aβ can induce the production of IL-1β [96]. The migration of astrocytes to Aβ plaques is promoted by chemokines CCL2 and CCL3, which are generally released by activated microglial cells. Upregulation of CCL2 by LPS was found to promote synaptic impairment through recruiting activin A leading to loss of hippocampal plasticity (Figure 2).

Important pathways involved in the pathogenesis of AD include the amyloid cascade hypothesis, TAU hypothesis, cholinergic hypothesis, and excitotoxicity hypothesis. In the case of AD, CSF dysfunction is noticed even before cognitive decline. Activities of mTOR cause vascular irregularities in the brain decreasing cerebral blood flow which in turn sets up cognitive decline. The amyloid cascade hypothesis identifies the accumulation of Aβ plaques at different areas of CNS and related changes as the principal factor behind the development of AD [97]. TAU hypothesis proposed that hyperphosphorylation of TAU leads to form neurofibrillary tangles preventing its regular role of supporting axonal microtubules and subsequently plays a critical role in neurodegeneration [98]. Cholinergic hypothesis focuses on symptoms of cognitive decline and presents malfunctioning of cholinergic neurons as a pathophysiological factor towards initiation of AD [99]. Excitotoxicity refers to the unprecedented death of nerve cells due to the overstimulation of certain amino acid receptors [100]. A high concentration of glutamates activates N-methyl-d-aspartate and α-amino-3-hydroxy-5-methylisoxazole propionic acid receptors. As a result, voltage-gated calcium allows the entry of extracellular calcium into cells and thus a hindrance in neuronal energy metabolism leads to cell death.

## 4. Neuroinflammation

Inflammation is the response of our body system to eliminate both sources of cell injury along with the cell and tissue debris originating from the insult. The immune system activation observed in AD is labelled as neuroinflammation. Though classical signs of inflammation such as swelling, heat, and pain are absent in brain inflammation, it characteristically involves increased monocytes and glial macrophage cells [31]. During the initial phase of neurodegeneration, immune reactions are triggered through the activation of macrophages (mainly M2 and sometimes M1) [101]. These activated macrophages secrete chemical messengers in interneuronal communications and develop autoimmune neurotoxicity including those reactions that lead to neuroinflammation and the escalation of AD. Activated cells strongly produce inflammatory mediators such as pro-inflammatory cytokines, chemokines, macrophage inflammatory proteins, monocyte chemo-attractant proteins, prostaglandins, leukotrienes, thromboxanes, coagulation factors, ROS (and other radicals), nitric oxide, complement factors, proteases, protease inhibitors, pentraxins, and C-reactive protein. Upregulated immunoinflammatory events play important roles in the pathogenesis of AD.

Chronic neuroinflammation (immune response to the formation of Aβ peptides and neurofibrillary tangles) is characterized by persistent activation of microglia and release of inflammatory mediators. Hence, an inflammatory cycle is perpetuated since microglia and astrocytes are constantly activated, leading to a further increase in the levels of cytokines and chemokines. These mediators, in turn, alter APP processing encourage the formation of Aβ plaques. These alterations also result in reduced production of neuroprotective sAPPα. Senile plaques activate the complement system resulting in inflammation within CNS. Thus, neuroinflammation-mediated tissue damage initiates the degeneration process. During the early stages of AD, neuroinflammation leads to the entry of PNS cells with chemokine receptors into the brain crossing BBB [102]. As a result of Aβ deposition, chemokines e.g., CCL2, IL-8, CXCL10, CCL5 are released from PNS.

Aβ plaques containing dystrophic neuritis, activated microglia, and reactive astrocytes that along with released inflammatory mediators contribute to neuronal dystrophy. Inflammatory mediators and activated glial cells together kill neighboring neurons and encourage amyloidogenic processing of APP. Nuclear receptor binding factor 2 (NRBF2) is a key factor for maintaining autophagic degradation of APP and production of Aβ by controlling maturation of APP-containing vesicles through the interaction of APP with CCZ1-MON1A-RAB7 module [103,104]. The inability of CNS phagocytes to clear Aβ plaques and upregulated formation of plaques as a result of chronic neuroinflammation play instrumental roles in AD [105]. In agreement with this, in a cohort study, Taipa and colleagues reported elevated levels of eotaxin, IL-1 receptor antagonist (IL-1ra), IL-4, IL-7, IL-8, IL-9, IL-10, IL-15, TNF-α, granulocyte colony-stimulating factor (GCSF), MCP1, and platelet-derived growth factor in CSF of AD patients in comparison with non-demented controls [40]. The same study also reported inverse relations between CSF levels of IL-1β, IL-4, IL-6, IL-9, IL-17A, IFN-γ, basic FGF/FGF2, GCSF, GMCSF, and MIP1β with AD progression [40]. In this section, we reviewed the roles of several neuroinflammatory factors including pro- and anti-inflammatory cytokines, APP and TAU proteins, glial cells, advanced glycation end products, and complement systems in the pathogenesis and development of AD.

### 4.1. Pro-Inflammatory Cytokines

**Figure 3:**
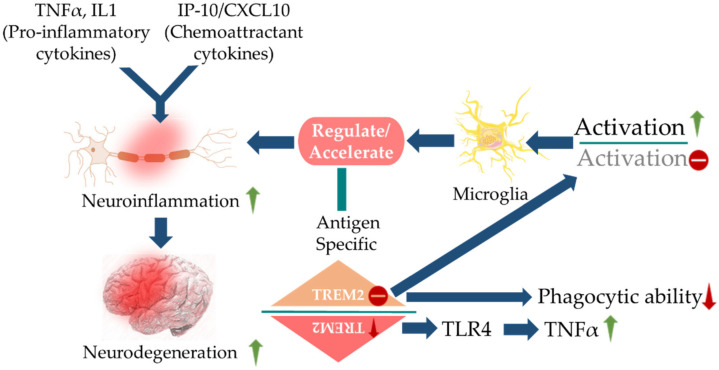
Pro-inflammatory cytokines and chemoattractant cytokines are key characteristic of neuroinflammation that can be acquired by the activation of microglia and can escalate neurodegeneration. Abnormalities in the TREM2 variant lead to defective microglial activation and decrease its phagocytic ability. The blue arrows (**↑**) indicate downstream cellular events, upward green arrows (**↑**) indicate upregulation, downward red arrows (↓) indicate down-regulation, and minus signs (**−**) indicate decreased activity.

Cytokines are secreted by glial cells around Aβ plaques. Disturbances in inflammatory and immune pathways in AD have been strongly associated with altered levels of some acute-phase proteins and pro-inflammatory cytokines in the blood, CSF, and brains. Aβ peptides can directly trigger the expression of several pro-inflammatory cytokines such as IL-1β, IL-6, TNF-α, and IFN-γ by glial cells. Pro-inflammatory cytokines like MMIF, YKL40, TNFs, and their receptors, sTREM2 are clearly engaged in TAU pathology and in the aging process [32]. Additionally, IL-15, MCP-1, VEGFR-1, sICAM1, sVCAM-1, and VEGF-D are found to be associated with TAU pathology and correlate with CSF TAU level [106]. Pro-inflammatory cytokines were found to induce indoleamine 2,3 dioxygenase to increase blood levels of quinolinic acid, a neurotoxic factor [107]. Pro-inflammatory cytokines, in conjugation with chemoattractants endorse neurodegeneration via promoting neuroinflammation, which can be triggered by the activation of defective microglia. TREM2 deficiency strongly triggers neuroinflammation via potentiating microglial activation and reducing microglia-mediated Aβ phagocytosis. TREM2 deficiency is also associated with activation of inflammatory markers, such as TNF-α through a TLR-dependent pathway (Figure 3).

High levels of pro-inflammatory cytokines, such as IL-1β, IL-6, and TNF-α, have been detected in the brain of AD subjects [108]. Pro-inflammatory molecules produced by the reactive astrocytes can elevate the expression of secretases in neurons, enhancing the production of Aβ and activating microglia to produce inflammatory factors [109]. In transgenic mice model, pro-inflammatory cytokines viz. IL-1 β, TNF-α, IL-6, IL-12, and IL-23 have also been found to correlate with Aβ load [110].

IL-1α and IL-1β are known to initiate cell activation upon binding with cell membrane receptors. Physiologically, an elevated level of IL-1β is a characteristic feature of brain parenchymal cells immediately after injury [111], while IL-1 hastens neuronal degeneration by increasing the production of IL-6 and the activity of iNOS. In addition to that, IL-1 is also responsible for enhanced acetylcholinesterase activity, activation of astrocytes and microglial cells, expression of S100β, production of macrophage colony-stimulating factor (MCSF), and further additional production of IL-1. IL-6 is a major player in host inflammatory response. IL-6 displays neurotrophic effects by activating microglia, promoting astrogliosis, and stimulating the production of acute-phase proteins. IFN-γ endorses TNFs and NO activities. TNF-α centrally regulates cytokine activities during inflammatory response through regulating an autocrine cascade of production of IL-1 and TNF-α from glial cells. In the AD brain, IL-1 regulates APP processing. In an experiment, rat cortical glial cells presented themselves with increased IL-6 mRNA on being exposed to the first 105 carboxy-terminal amino acids of APP [112]. Dose-dependent increments were also observed in levels of IL-1, IL-6, TNF-α, MIP-1α, and MCP-1 in glial cells on exposure to Aβ peptides [74].

**Figure 4:**
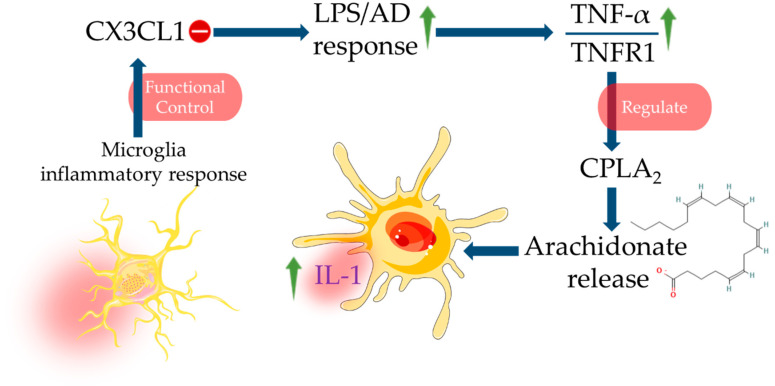
Absence of CX3CL1 upregulates LPS response leading to increase in TNF-α expression. TNFR1 in turn regulates CPLA2 to stimulate arachidonate release. Arachidonate release can further lead to IL-1 release from macrophages. The blue arrows (**↑**) indicate downstream cellular events, upward green arrows (**↑**) indicate upregulation, and minus sign (**−**) indicates decreased activity.

In contrast, IL-1ra, IL-4, IL-10, IL-11, IL-13, TGF-β act as anti-inflammatory cytokines, specific receptors for IL-1, TNF-α, and IL-18 act as inhibitors of pro-inflammatory cytokines. Anti-inflammatory cytokines belonging to Th2 and Th3 cell subsets exert a protective effect against AD by counteracting the effects of pro-inflammatory cytokines [80]. Of note, TGF- β, produced by Th3 cells is capable of ameliorating Aβ-induced cytotoxicity both in vivo and in vitro; while, deficiency of TGF-β1 promotes accumulation of Aβ peptides and formation of neurofibrillary tangles [113]. Dysregulation of the balance between pro-inflammatory and anti-inflammatory cytokines in the favor of pro-inflammatory cytokines leads to a cycle of further cytokine production, cytokine synergism, and cellular activation. It has been shown that an absence of chemokine (CX3CL1) can increase TNF-α and TNFR1 expression by intensifying LPS action, which simultaneously triggers the release of other pro-inflammatory cytokines like IL-1 by macrophages mediated through enhanced arachidonate release. Microglial hyperactivation can lead to CX3CL1 impairment in the brain, which ultimately impacts by amplifying and worsening the neuroinflammatory conditions (Figure 4).

### 4.2. Anti-Inflammatory Cytokines

Interestingly IL-4, IL-10, and IL-13 can suppress pro-inflammatory cytokine genes e.g., IL-1, TNFs, and chemokines. IL-1ra directly antagonizes the activities of IL-1α and IL-1β by competitive inhibition. Experimental results suggest that IL-1ra suppresses IL-1β-induced TNF-α production and iNOS expression in astrocytes by preferentially binding with IL-1R1 [29]. In addition to protecting against IL-1β-induced neurotoxicity, IL-1ra also attenuates neuronal damage caused by ischaemic excitations. IL-4 can suppress pro-inflammatory cytokines such as IL-1, TNF-α, IL-6, IL-8, and MIP-1α by inhibiting their expressions. Further IL-4 is associated with increased production of IL-1ra and inhibition of IFN-γ leading to a decrease in TNF-α and NO. IL-10, acting through specific cell surface receptors reduces the synthesis of IL-1 and TNF-α. IL-10 also inhibits TNF-α, IL-1, IL-6, IL-8, IL-12, GMSF, MIP-1α, and MIP-2α. Secretion of pro-inflammatory cytokines by glial cells is halted on pre-exposure to IL-10. IL-10 has been hypothesized to exert the actions by suppressing cytokine receptor expression, inhibiting receptor activation, while TGF-β has been shown to impede the production of IL-2, IFN-γ, and TNFs. Of note, three mammalian isoforms of TGF-β i.e., TGF-β1, TGF-β2, and TGF-β3 are prevalent within the CNS. As a result of this, TGF-β is associated with a plethora of activities including microglial activation to inflammatory response, astrocytes, and regulation of COX-2 and APP. Interestingly, elevated levels of TGF-β1 and TGF-β2 have been observed in CSF and blood of AD patients [114,115].

### 4.3. APP Protein

APP is a transmembrane protein present in the cell membrane of all neurons. Under normal conditions, α-secretase and γ-secretase cleave APP into three fragments which in turn get digested via proteosomes (non-amyloidogenic pathway). During the initial phases of AD, the amyloidogenic pathway takes over and β-secretase becomes involved in the process in place of α-secretase [116]. The α-secretase activity is exerted by three members of the ADAM family *viz.* ADAM9, ADAM10, and ADAM17/TACE. The β-secretase activity has been mainly attributed to the β-site APP cleaving enzyme. The γ-secretase complex comprises presenilin (PSEN), nicastrin, anterior pharynx defective-1 (APH-1), and presenilin enhancer-2 (Pen-2). The amyloidogenic pathway predominantly gives rise to fragments like sAPPβ, APP intracellular domain (AICD), and Aβ peptide spanning from 1-40 amino acid residues. It further exacerbates AD symptoms as these abnormal fragments are not naturally digested resulting in extracellular accumulation of aggregates or plaques of those fragments. Eventually, these senile plaques are termed Aβ peptides or Aβ lipoproteins. These senile plaques, in general, lead to neurotoxicity, apoptosis, oxidative stress, and neuroinflammation. In addition to generating inflammatory responses, Aβ also causes mechanical disruption in synaptic transmission [117].

### 4.4. TAU

TAU protein stabilizes microtubules which are very important for the cytoskeletal integrity of a cell. They reside throughout the axon to aid transport proteins to move nutrients and neurotransmitters. Microtubules lose their structure in absence of TAU and break apart. When β-secretase becomes more active than α-secretase, thus a high amount of Aβ is produced that in turn, causes hyperpolarisation of TAU protein through excessive phosphorylation of TAU [118]. On hyperpolarisation, TAU protein starts aggregating with each other. Unlike senile plaques, TAU clumps stay inside neuronal cells. As a consequence of this, the cytoskeleton starts to fall apart that hampers axonal transport. Neurotransmitter transport from soma to synaptic bud becomes affected and neuronal function decreases. Not only neurotransmitters, but the flow of nutrients inside the longest cell of the body also suffers, and gradually axons and dendrons start to degenerate. As a result of this, the cluster of such neurons forms neurofibrillary tangles. Cytokines with kinase activity on TAU include cyclin-dependent kinase 5 (CDK5), glycogen synthase kinase-3β (GSK-3β), and p38 mitogen-activated protein kinases (p38-MAPK) [119].

In AD, these TAU-led neurofibrillary tangles have been observed to be further propagated through the toxicity presented by Aβ plaque accumulation and loss of cholinergic neurons in rat basal forebrain primary septal culture [120]. Additionally, Aβ was found to prevent microtubule binding in primary cultures of fetal rat hippocampal neurons. While in the human cortical neurons induced hyperphosphorylation of TAU at Ser-202 and Ser-396 was found to be accumulated in the somatodendritic compartment of Aβ-treated neurons [121].

The constituents of axonal projections in the mammalian brain are neurofilaments that form side projections of carboxy-terminals from the core filament, believed to be heavily phosphorylated; while TAU-embellished microtubules are also known to be differentially phosphorylated. The α- and β-globulin subunits that constitute axonal microtubules are formed by the energy-consuming nucleation process. An energy-expensive neuro-process would require optimal active mitochondria to properly conduct impulse. Hyperphosphorylation of TAU has been credited to play an active role in the impairment of axonal support functioning that optimizes interneuronal communications amongst associated organelles. The oxidative stress in AD brains also may lead to hyperphosphorylation of TAU. Of note, where the absence of superoxide dismutase (SOD) was observed to increase oxidation damage from ROS, an escalation of Ser-36 phospho-TAU was revealed in treatments of SOD-null mice. Untreated mice did not survive past one week, reflecting SOD deficiency was, therefore, deleterious [122].

### 4.5. Glial Cells

Progress in AD-related research has revealed the important roles of glial cells including the astrocytes, microglia, NG2 glia, and oligodendrocytes that contribute to the pathogenesis of the disease [123]. Astrocytes and microglia participate by functioning as effector cells to release cytokines by somehow failing to live up to their homeostatic functions. NG2 glia, a novel and distinct class of glial cells in CNS are responsible for myelination and remyelination of axons thus playing a vital role in high-speed nerve impulse transport and cognition [124]. It is interesting to note that amyloid peptides and their precursor APP protein act as glial activators. Disruption of the APP gene and its proteolytic products delay and decrease amyloid-dependent microglial activation.

Astrocytes are star-shaped glial cells in CNS involved in energy reserves, regulation of extracellular ions, as well as the clearance, metabolism of neurotransmitters, and modulation of oxidative stress. Among the notable neurotransmitters, glutamate is released during neuroinflammatory conditions mainly which in the long-term is proved to be toxic to neurons via the excitotoxicity pathway. Of note, astrocytes can take up glutamate and recycle it to neurons after transforming into glutamine, an amino acid [125]. In the AD, Aβ peptides decrease uptake of glutamate, resulting in increasing redox insult. Interestingly, alongside the neuroprotective activities of astrocytes through Aβ clearance and degradation, they could also be a source of Aβ owing to their overexpression of beta-secretase 1 (BACE1) in response to chronic stress [126].

The migration of astrocytes to Aβ plaques is promoted by chemokines CCL2 and CCL3, which are released by activated microglial cells. In an experimental model, mouse astrocytes plated on amyloid-rich brain sections from APP transgenic mice have been found to reduce amyloids [45]. Of note, astrocytes respond to CNS insults through a process named reactive astrogliosis, an early pathological feature of AD, and can represent a response to the accumulation of Aβ and/or to the increasing number of degenerating neurons [127]. Astrocytes can be stimulated by oxidative stress, free saturated fatty acids, pathogens, and LPSs. Additionally, contrary to quiescent astrocytes, reactive astrocytes can produce cytokines, such as TNF-α, IFN-γ, and ILs [41]. IFN-β, TNF-α, and IL-1β induce the generation of Aβ in primary human astrocytes and astrocytoma cells. Astrogliosis is also characterized by a high level of the astrocyte marker glial fibrillary acidic protein (GFAP). The latter occurs around Aβ deposits both in the brain parenchyma and in the cerebral microvasculature. Senile plaques are associated with GFAP-positive activated astrocytes. In various neuropathological states, the increased expression of GFAP corresponds to the severity of astroglial activation [128]. Microglial cells and astrocytes express pathogen recognition receptors e.g., TLRs, integrin α6β1, A1, CD36, CD47, CD14 to act as class A scavenger receptors through DAMP [80].

Oligodendrocytes, under the influence of NG2 cells, are responsible for myelin sheath generation around axons. A study concluded that Aβ peptides induce local translation of myelin basic protein 18.5 kDa isoform in distal cell processes [129]. It is interesting to note that Aβ oligomers modulate the expression of myelin basic protein with the help of the integrin β1 receptor, Src-family kinase Fyn, and Ca^2+^/CaMKII. The pharmacological inhibition of Fyn kinase was found to attenuate oligodendrocyte differentiation and survival induced by Aβ. Interestingly, in ex vivo organotypic cerebellar slices, Aβ caused upregulation of myelin basic protein through Fyn kinase and modulated oligodendrocyte population dynamics by inducing cell proliferation and differentiation [129]. Application of Aβ oligomers to cerebellar organotypic slices, enhance remyelination and oligodendrocyte lineage recovery was suggested in the case of lysolecithin-induced demyelination.

### 4.6. Advanced Glycation End Products

Advanced glycation end products mediate crosslinking of certain proteins resulting in age-related decline in cognition and other cellular functions [130]. RAGE (receptor for advanced glycation end-products), a ligand for both Aβ and S100B is also associated with the activity [131]. In hyperglycaemic patients, unusual glucose metabolism and oxidative stress aggravate the activities of advanced glycation end-products [132]. This may be correlated with the notion that excess dietary carbohydrates and deficient cholesterol may lead to AD development. Intracellular neurofibrillary tangles and extracellular senile plaques serve as substrates for glycation. Advanced glycation end products induce the production of ROS and cytokines through activation of microglial RAGE leading to engagement of nuclear factor kappa B (NF-κB) [133]. It has been clinically observed that low dietary intake of advanced glycation end products is directly related to reduced oxidative stress and inflammation that can further exacerbate AD symptoms [134,135].

### 4.7. Complement System

At an early stage of AD, Aβ peptides activate the complement system. The complement system works as a part of the immune system to remove unwanted bodies through antibody-mediated phagocytosis. In course of doing this, complementary proteins interact with cell surface receptors to promote an inflammatory response in the host system. Complement system attacks and destroys invaders in four steps *viz.* recognition, opsonization, inflammatory stimulation, and killing. In the human brain, astrocytes are the major center of complement activity. Astrocytes can synthesize complement proteins including C1-C9, regulatory factors B, D, H, I, and complement receptors namely C1qR, C3aR, and C5aR locally to defend through both classical and alternative pathways [74]. Microglia also supports phagocytosis by expressing C1q, C3 proteins, and C1qR, CR3, and C5aR receptors [136]. Apart from neuroglia, neurons also express regulatory factors H, S, and receptors C1qR, C3aR, and C5aR. Complement protein C1q affects the formation of Aβ plaques containing β-sheet structures [137]. In transgenic AD mice, inhibition of the complement system by C3-knockout resulted in the increased formation of Aβ plaques. These results have further supported a neuroprotective role of the complement system [137,138,139].

## 5. MMIFs in AD: Pathogenic or Protective?

MMIF, also termed as a glycosylation inhibiting factor, is classified as a pro-inflammatory cytokine is an important regulator of innate immunity. Expression of MMIF correlates with expression of VEGF in CNS [140,141]. Interestingly, glucocorticoids stimulate the secretion of MMIF, whereas glucocorticoids are known to suppress most of the other cytokines. Thus, MMIF acts against the general anti-inflammatory response of glucocorticoids. There exists a debate on whether endogenous MMIFs support or counter the pathogenesis of AD. Enhanced MMIFs have been reported in mouse models of neurodegenerative disorders [80,142]. Again, several studies reported that MMIF-knockdown in mutant mice has resulted in the acceleration of neurodegenerative disorders [143,144]. MMIFs have also been reported to regulate neuroinflammation and autophagy in the favor of neuroprotection [144,145,146].

MMIF has a notable function in controlling the synthesis and release of TNF-α, IL-1, and other cytokines. MMIF is also involved in macrophage functions such as phagocytosis and tumoricidal activities. On the other note, a brain insulin-resistant state arises due to prolonged exposure of cortical neurons to high concentrations of insulin. MMIF contributes to this insulin-resistant state through inhibition of Akt phosphorylation [147]. In some cases, a structural homolog of MMIF, D-dopachrome tautomerase (MIF-2) exhibits synergistic activities in combination with MMIF [148]. Moreover, MMIF and fragments of senile plaques display similar neurotoxicity patterns [149]. The study also reported enhanced MMIF levels in CSF of AD patients [149]. In silico studies further suggest that MMIF may be involved in neuronal apoptosis during AD [150]. However, it is interesting to note that Popp and colleagues earlier did not find any difference in MMIF levels of AD patients with mild, moderate, and severe dementia [151]. Conclusively, we can say that imbalance between oxidized and reduced isoforms of MMIF is the key to regulate the switch to either a diseased or normal state [151].

## 6. Choroid Plexus Growth Factors and AD

The growth-promoting properties of APP, along with other growth factors, play vital roles in the development of AD. The choroid plexus supports neuronal function by secreting CSF. VEGF and FGF can be found in epithelial cells of the choroid plexus. It is rich in various proteins and their receptors. Proteins include FGF-2, TGF-α, and TGF-β along with mRNA expressions for TGF-β, IGF-II, FGF-2, and NGF receptors. The choroid plexus also contains receptor binding sites for FGF-7, keratinocyte growth factor, IGF-1, and IGF-2. Blood-CSF barrier made up of epithelial cells and tight junctions at the choroid plexus allow selective passage of materials into the brain. FGF-2 has been reported to increase in brain parenchyma of AD patients. Moreover, infusion of FGF-2 in rats has resulted in hydrocephalus ex vacuo, which is a clinical feature of AD [152]. It is important to note that improper CSF circulation and impaired clearance of CSF may give rise to dementia and neurodegeneration due to lack of nutrition to CNS cells and enhanced toxic accumulations within CSF. In this section, we shed light on the specific roles of VEGF and FGF growth factors in the development of AD.

### 6.1. Vascular Endothelial Growth Factors (VEGFs)

VEGFs and their receptors have been reported to localize at the area with lesions and AD-related developments. Different isoforms of VEGF act as pro-inflammatory cytokines, which increase endothelial cell permeability, induce the expression of endothelial cell adhesion molecules and act as monocyte chemoattractants [153]. VEGF is involved in the regulation of GLUT1 and tissue thromboplastin, which in turn regulate vascular pathologies of AD. GLUT1, present in BBB mediates glucose transport into the brain and reduced expression of GLUT1 is relatable with aggravated AD conditions. Tissue thromboplastin and derived factors play a pro-inflammatory role leading to vascular dementia [154]. AD patients tend to present with enhanced VEGF activity within reactive astrocytes [155]. Rats subjected to cerebral ischemia displayed increased perivascular VEGF reactivity in the clusters of reactive astrocytes [156].

### 6.2. Fibroblast Growth Factors (FGF)

FGFs are circulatory proteins that play important roles in the activation of cell surface receptors. Around 23 FGF subtypes have been known to exert distinct functions to date [157]. Acidic FGF-1 and basic FGF-2, among eight other FGF family proteins, act through four families of FGF receptors. However, FGF-11-14 does not act through FGF receptors.

FGF-1 and FGF-2 are more potent angiogenic factors than VEGF [52]. Within CNS, FGFs play important roles in the proliferation and differentiation of neuronal stem cells including neurogenesis and axonal growth. FGFs also support the self-renewal of radial glial cells. FGF-8 is a vital player for the proper functioning of the cerebral cortex. Increased levels of FGF-2 have reportedly been associated with AD brain leading to enlargement of ventricles [158]. FGFs regulate not only neuronal stem cells but also adult neurogenesis. Additionally, the maintenance and survival of neurons throughout their life depend greatly on FGF-2. Synaptic plasticity, to some extent, is controlled by FGF-1 and FGF-2. Thus, the conduction of nerve impulses through axons and synapses for proper cognition is dependent upon FGFs. Belluardo and colleagues demonstrated that upregulation of FGF-2 can successfully prevent neuronal loss in cortical and hippocampal regions of the brain [159]. In the rat models, FGF-21 has been found to ameliorate senile plaques-mediated neurodegeneration [160]. The effects were achieved via minimizing oxidative stress through PP2A/MAPK/HIF-1α-mediated pathways [160].

## 7. Neurotrophic Factors

Neurotrophic growth factors produced by neural stem cells are involved in the differentiation of cells and cell survival. Neurotrophic growth factors consist of NGFs, GDNF, neurokines, and non-neuronal growth factors. NGF is probably the most discussed neurotrophic growth factor/neuropeptide that involves in growth regulation, maintenance, proliferation, and survival of certain target neurons. NGF was the first neurotrophin to be discovered followed by BDNF, neurotrophin-3, neurotrophin-4/5, and neurotrophin-6 [72]. Neurotrophins bind to cognate TrK receptors and p75NTR. The low-affinity p75NTR can bind with all neurotrophin family members. Neurokines and cytokines related to IL-6 bind to cell surface receptor complexes, which share a common structural organization. The four ligands interchangeably employ two distinct receptor subunits, leukemia inhibitory factor receptor b (LIFRb) and gp130; some employ a ligand-specific α subunit [76].

NGF exhibits protective action over cholinergic neurodegeneration. NGF can influence APP processing towards the non-amyloidogenic pathway via protein kinase C-coupled M1 and M3 receptors. Interestingly, NGFs are upregulated in AD brain and CSF, while NGF receptor TrKA is downregulated [74]. BDNFs alone and in chimeric combination with NGF have been found to protect cholinergic neurons in prosencephalon [58]. Interestingly, AD brains have been diagnosed with decreased levels of mRNAs for BDNFs but normal levels of mRNAs for NGF and neurotrophin-3 [161]. In the AD brain, astrogliosis may contribute to increasing NGF and reducing TrKA in the cortex and nucleus basalis. Vinculin-dependent adhesions are central to the functioning of NGF to promote axonal outgrowth. Vinculin-dependent coupling regulates the level of myosin needed for NGF stimulation. The role of NGF as a growth factor amongst a bouquet of proteins is paramount in cognitive processes that may be involved in the survival and phosphorylation of fibrils in axons, that are involved in AD and other chronic diseases closely related to AD [56].

## 8. Hematopoietic Growth Factors

Apart from controlling hematopoiesis in blood progenitor cells, hematopoietic growth factors such as IL-3, GCSF, GMCSF, MCSF, and erythropoietin play vital roles in the functional activation of all mature cells. In the biological and pathological role of the immune system, the immune system achieves its role by cells that encapsulate it as a whole. Such cells originate from hematopoietic stem cells in the bone marrow by a blood-forming process of hematopoiesis that gives rise to myeloid progenitor cells and lymphoid progenitor cells [162]. Myeloid progenitor cells constitute megakaryocytes, erythrocytes, mast cells, and myeloblast. The myeloblast cells differentiate into immune cells, such as basophil, neutrophil, eosinophil, and monocytes. Of the subset of the myoblast cells are the monocytes that later develop into macrophages, which play an initiating part in immune system responses that counter foreign material, pathogens, and compromised cells in the CNS.

**Figure 5:**
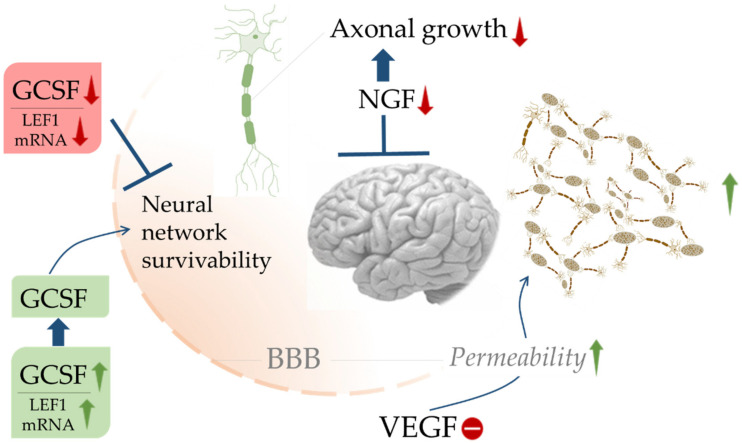
Schematic representation of functional control across BBB by hematopoietic growth factors. The blue arrows (**↑**) indicate downstream cellular events, blue lines (T) indicate restriction, upward green arrows (**↑**) indicate upregulation, downward red arrows (**↓**) indicate down-regulation, and minus sign (**−**) indicates decreased activity.

Hematopoietic growth factors are important contributors to brain marrow for neuropoiesis. They can prevent neuronal death to some extent. Jin and colleagues have pointed out enhanced neurogenesis during AD progression [163], though many pose doubts on the marker doublecortin [164,165]. In a mouse model, GCSF has been observed to restore cognition by restoring acetylcholine levels [61]. The survivability of neural networks in the brain largely depends on GCSF and LEF1 availability, which enter through the BBB and promote their survivability. VEGF increases BBB permeability; however, a defective VEGF expression can trigger immunoreactivity, which is a characteristic feature in AD (Figure 5). Stem cell factors, in combination with receptor c-kit, stimulate neurogenesis [62]. The lower level of stem cell factor in blood and CSF were observed to accelerate cognitive decline during AD [63]. Increased levels of angiopoietins 1 and 2 indicate a cognitive decline in AD. In the mouse models, angiopoietin 1 accelerates AD via FOXA2/PEN2/APP-dependent pathway [166]. Increased neurogenesis, anti-apoptotic influences, and mobilization of microglia contribute to brain repair involving hematopoietic growth factors.

## 9. Potential Strategies Involving Cytokines for Management of AD

AD affects millions of individuals worldwide among the aging population, yet no therapeutic intervention is available to stop and eliminate this disorder. Neuropathological hallmarks of AD are extracellular deposits of Aβ peptides assembled in plaques, intraneuronal accumulation of hyperphosphorylated TAU protein forming neurofibrillary tangles, and chronic neuroinflammation. No absolute cure for AD is available so far [167].

Among the available therapeutic options against AD, cholinesterase inhibitors and NMDA antagonists display moderate relief in the case of AD. Donepezil, an inhibitor of acetylcholinesterase improved cognitive conditions in AD and increased BDNFs [168]. Pharmacotherapy against Aβ and TAU has yielded limited success only. Treatment with β-sheet breaker peptides results in reduced brain inflammation by disrupting amyloids [169]. RAGE/NF-κB axis could be a potential therapeutic target in AD [170]. Some dietary nutraceuticals display inhibitory effects on the formation of advanced glycation end-products [171]. Resveratrol has been found to modulate levels of Aβ and certain inflammatory markers in AD patients [172]. Luteolin can play a prophylactic role against AD [173]. Additionally, moderate activation of microglia is thought to have beneficial effects in removing neurotoxins, cellular debris, and dying cells or in promoting neuronal survival. Since MMIF is augmented in AD, measuring blood and CSF levels of MMIF may represent a diagnostic biomarker useful both for diagnosis and therapeutic monitoring of the disease [174]. Moderate activation of microglia by acute neuroinflammation is thought to have beneficial effects in removing neurotoxins, cellular debris, or dying cells and also in promoting neuronal survival [175]. IL-1ra, a glycosylated protein antagonizes the cell activating action of IL-1. Furthermore, TNF-α has been reported to possess neuroprotective effects [176]. TGF-β is capable of converting an active site of inflammation into one dominated by reparations [177]. Kitazawa et al. described that blocking IL-1 signaling in 3xtg AD mice with an IL-1 receptor blocking antibody was beneficial since it leads to a decrease in certain Aβ fibrillar forms and plaques [27].

It has been suggested that a blockade of the ongoing inflammatory processes may delay the progression of AD [178]. Studies suggest lesser incidents of developing AD in arthritis patients receiving NSAIDs, regularly [179,180]. The fact that COX-2 mRNA is upregulated in the AD brain further supports this claim. Therefore, receptors for hematopoietic growth factors expressed on neurons provide novel targets for drug discovery in the search for agents that can reverse the progression of AD.

It is interesting to observe that peripheral phagocytes can effectively clear plaques and therapeutic strategies aiming at favoring the recruitment of these cells into the CNS are actively being pursued [80]. In a mouse model, the BDNFs have improved AD conditions by delaying synaptic loss, improving cell signaling, and enhancing cognition and spatial learning [181]. GCSF and analogs have proven neuroprotective activity, which may possibly be used therapeutically. In vivo intraperitoneal VEGF administration reduced cognitive impairment in a mice model of AD [53]. As discussed earlier, NGFs are potential candidates for significant improvement of cognitive functions. Biogenetic exosome-mediated activation of microglia and deregulation of microRNA can be useful to fight against neuroinflammation [182]. Erythropoietin, together with NF-κB can prevent neuronal injury triggered by Aβ toxicity [183]. Inhibitors of TNF-α have exhibited potential promise to slow down the progress of AD-associated cognitive decline [183]. Experimentally delivered mature NGFs into the AD brain showed potential for improving AD condition [56]. ApoE4-centric treatment approaches are gaining interest in recent times since ApoE4 is involved in more than 50% of AD cases [184]. M2 microglia are generally engaged in the restoration of homeostatic balance after an inflammatory insult by releasing anti-inflammatory factors. Thus, the therapeutic promise is there to prevent and treat neuroinflammation with protective functions of microglia [185,186,187]. Another potential strategy might be to inhibit BACE1 to reduce the production of Aβ, however, clinical success is yet to be achieved [188]. Recently, multitarget-directed ligand-based treatment strategies have started to evolve centering on inhibition of GSK-3β, a crucial enzyme for TAU hyperphosphorylation, and some other CNS-specific signaling pathways [119]. Nowadays, in the war against AD and associated disorders, researchers are focusing more on regulating neurotransmitters, lipid metabolism, autophagy, circadian rhythm, gene therapy, etc. [189].

## 10. Conclusions

In this review, ample evidence reflects the potential roles of cytokines and growth factors in the pathogenesis of AD or pathologically related to AD-like neurodegenerative conditions. It helps us to understand the propensities and action of cytokines and growth factors regulating their effects on neurons upon neurodegeneration. Altogether, evidence evinced in previous research on the rather novel concentration on the topic of cytokines in neuroimmune system responses and their role in inflammation. These two factors possibly preceding neurotoxicity and intrathecal generation of immune molecules and cytokine-producing cells show that cytokines mediate and even activate innate neuroimmune agents. Cytokines regulate the response of pro-inflammatory and anti-inflammatory signals to maintain CNS machinery homeostasis [190]. Pro-inflammatory cytokines induce inflammation in AD and AD-like pathogenesis in response to the apoptotic scenarios. Some growth factors are implicated in the expression of cytokinetic reactions to activate microglia that cause inflammation in AD. Cytokines and growth factors such as NGF, VEGF, TNF-α, and IL-1 additionally impact intricate molecular processes necessary for balance and homeostasis in cognitive mechanisms. To conclude, there exists ample scope of improvement regarding clinically useful strategies to mitigate AD.

## Figures and Tables

**Table 1:** Stepwise progression of AD.

Serial. No.	Stages	Pathological Symptoms
1	Early onset AD/MCI	Impairment of non-memory features of cognition, difficulty in word finding, decline in reasoning/judgement.
2	Mild AD	Loss of spontaneity, memory loss, anxiety, aggression, restlessness, altered personality, misplacing items.
3	Moderate AD	Confusion, attention deficit, continuous cognition problems, impulsive behavior, delusion, paranoia, hallucination, recognition problem.
4	Severe AD	Severe dementia, continued cognitive decline, seizures, functional limitations, lack of bowel/bladder control, weight loss, skin infection, swallowing difficulty, enhanced sleep time, brain atrophy.

**Table 2:** Changes mediated by cytokines and growth factors within CNS.

Serial No.	Mediators	Functions	References
1	IL-1α	Increases α-secretase, decreases amyloidogenic processing, increases sAPPα	[24,26,27]
2	IL-1β	Increases APP mRNA, increases α-secretase and γ-secretase, downregulates β-secretase, upregulates TAU mRNA	[28,29,30]
3	IL-4	Upregulates Aβ production, increases p-TAU	[30,31]
4	IL-6	Upregulates APP mRNA, increases p-TAU	[10,32]
5	IL-8/CXCL8	Upregulates γ-secretase activity by increasing substrates C83 and C99	[33,34]
6	IL-10	Favors Aβ deposition	[10,35,36]
7	IL-18	Increases APP, upregulates both β-secretase and γ-secretase, increases Aβ formation	[10,37,38]
8	TNF-α	Upregulates APP mRNA, upregulates both β-secretase and γ-secretase, increases sAPPβ	[10,36,39]
9	IFN-γ	Upregulates APP intracellular domains, upregulates both β-secretase and γ-secretase, increases Aβ deposition	[40,41,42,43]
10	TGF-β1	Increases APP mRNA, increases Aβ deposition	[10,42,43]
11	CCL2	Increases Aβ formation and deposition	[44,45]
12	CCL3	Upregulates β-secretase, increases C99, increases Aβ deposition	[45,46]
13	CCL5	Upregulates β-secretase, increase C99, increases Aβ deposition	[46,47]
14	CXCL10	Decreases Aβ deposition	[34,48]
15	CX3CL1	Decreased Aβ deposition, upregulated p-TAU	[49,50]
16	VEGF	Upregulates expressions of monocytes and macrophages, increases proliferation of endothelial cells	[51,52,53]
17	FGF	Attenuates Aβ related pathologies	[52,54]
18	NGF	Increases degeneration leads to loss of cholinergic nerve endings in cortex and hippocampus	[55,56]
19	BDNF	Upregulates sAPPα, promotes non-amyloidogenic pathway, astrocyte activation, improved memory performance	[57,58]
20	GDNF	Neuroprotection	[55,59]
21	GCSF	Induces neurogenesis	[60,61]
22	Stem cell factor	Maintains hematopoietic brain support, neurogenesis	[62,63]
23	SDF	Neurogenesis, inflammatory disruption of BBB	[64,65]
24	CXCR4	Ligand for SDF-1	[64,66]
25	Angiopoeitins	Angiopoeitin-1 prevents neuronal apoptosis, Angiopoeitin-2 promotes neurogenesis via migration of neural progenitor cells	[67,68,69]
26	Neurotrophin-3	Upregulates neuronal apoptosis inhibitory protein 1, limits cleavage of caspases 3, 8 and 9	[70,71]
27	Neurotrophin-4	Regulates TAU dephosphorylation	[70,72]
28	TrKA	Receptor protein for β-NGF	[73,74]
29	TrKB	Receptor protein for brain derived neurotrophic factor and neurotrophins	[73,75]
30	TrKC	Receptor protein for neurotrophin-3	[73,76]
31	p75	Neurotrophin receptor protein, regulates phosphorylation of TAU	[71,72]

## Data Availability

Not applicable.

## Abbreviations

AD	Alzheimer’s disease
ADAM	A disintegrin and metalloprotease
AICD	APP intracellular domain
APH-1	Anterior pharynx defective-1
ApoE	Apolipoprotein E
APP	Myloid precursor 69 protein
Aβ	Amyloid-beta
basic FGF/FGF2	Basic fibroblast growth factor
BBB	Blood-brain barrier
BDNF	Brain-derived neurotrophic factor
CDK5	Cyclin-dependent kinase 5
CNS	Central nervous system
CPLA2	Cytosolic phospholipase A2
CSF	Cerebrospinal fluid
DAMP	Danger-associated molecular pathways
GCSF	Granulocyte colony-stimulating factor
GDNF	Glial cell line-derived neurotrophic factor
GFAP	Glial fibrillary acidic protein
GMCSF	Granulocyte macrophage colony-stimulating factor
GSK-3β	Glycogen synthase kinase-3beta
IGF	Insulin-like growth factor
IL-1ra	IL-1 receptor antagonist
IL	Interleukin
INF	Interferon
LIFRb	Leukemia inhibitory factor receptor b
LPS	Lipopolysaccharide
MCI	Mild cognitive impairment
MCP1	Monocyte chemoattractant protein 1
MCSF	Macrophage colony-stimulating factor
MIIB	Myosin IIB
MIP	Macrophage inflammatory protein
MMIF	Macrophage migration inhibitory factor
NGF	Nerve growth factors
p38-MAPK	Mitogen-activated protein kinases
p75NTR	p75 neurotrophin receptor
Pen-2	Presenilin enhancer-2
PNS	Peripheral nervous system
PSEN	Presenilin
ROS	Reactive oxygen species
TACE	TNF-α converting enzyme
TGFβ	Transforming growth factor beta
TNFs	Tumor necrosis factors
TrK	Tropomyosin Receptor Kinases
VEGF	Vascular endothelial growth factor
